# Comparison of in vitro biocompatibility and antibacterial activity of two calcium silicate-based materials

**DOI:** 10.1007/s10856-021-06523-9

**Published:** 2021-04-26

**Authors:** Mingxiang Liu, Lu He, Hongyuan Wang, Wenpei Su, Hong Li

**Affiliations:** 1 Department of Endodontics, Beijing Stomatological Hospital, School of Stomatology, Capital Medical University, Beijing, 100050 China; 2 Affiliated Stomatology Hospital of Guangzhou Medical University, School of Stomatology, Guangzhou Medical University, Guangdong, 510140 China

## Abstract

This study is aimed at comparing and evaluating the biocompatibility and antibacterial activities of mineral trioxide aggregate (MTA) and iRoot BP Plus as novel retro-filling materials. Discs of both materials were prepared and incubated for 72 h to obtain material extracts in medium. Flow cytometry and the 3-(4,5-dimethylthiazol-2-yl)-2,5-diphenyltetrazolium bromide assay were used to assess the rate of apoptosis and proliferation of human periodontal ligament stem cells (hPDLSCs) when exposed to eluates of both materials. The expression levels of alkaline phosphatase, collagen type I, osteocalcin, Runt-related transcription factor-2, and Osterix were tested for evaluating the osteogenic differentiation of hPDLSCs. The antibacterial activities of both materials were compared by the direct contact test. The hPDLSCs stimulated by MTA or iRoot BP Plus eluates showed significantly higher cell viability than that of the control group with no eluates. No significant differences were observed among the percentages of necrotic and apoptotic cells stimulated by MTA and iRoot BP Plus eluates and the control group. The expression of all osteogenic differentiation markers of hPDLSCs in both experimental groups were significantly higher than those of the control group, while the increment values in MTA group were significantly higher than those of the iRoot BP Plus group. The antibacterial activity against *Enterococcus faecalis* showed no significant difference between MTA and iRoot BP Plus. Therefore, both materials may be suitable for retro-filling applications.

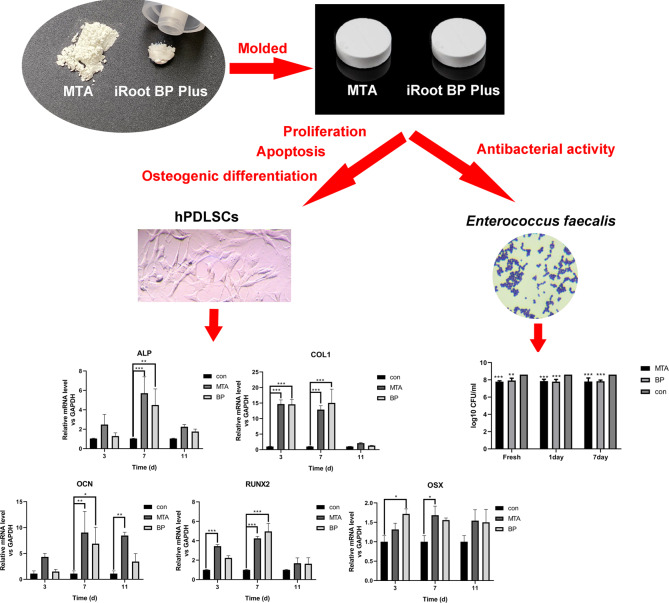

## Introduction

Among various oral biomaterials, mineral trioxide aggregate (MTA) has been widely used in apical surgery and endodontic therapy since the early 1990s because of its outstanding biological and physical properties [1]. MTA is a calcium silicate-based cement composed of tricalcium silicate, tricalcium oxides, tetracalcium aluminoferrite, and so on [2]. It has been successfully used for pulp capping, perforation repairing, root resorption, and apexification [3]. MTA exhibits high pH, good biocompatibility, bioactivity, osteoconductivity, cell viability promotion, and releases calcium hydroxide (Ca(OH)_2_) when exposed to water [1]. It has been shown to stimulate the release of cytokine in osteoblasts, which promotes hard tissue formation [4]. However, a lack of antibacterial activity against *Enterococcus faecalis* (*E.faecalis*) was detected using direct contact test (DCT) in a previous study [5]. Due to its high sealing and biocompatibility, MTA is considered the gold standard as a novel root-end filling material [6]. Nevertheless, it has several drawbacks, such as presence of toxic elements in its composition, long setting time, discoloration potential and difficulty in handling; consequently, improvements are necessary [7].

iRoot BP Plus is a new laboratory-synthesized bioceramic material used for oral surgery and permanent root canal repair [8]. iRoot BP Plus is composed of calcium silicate, calcium sulfate, zirconium oxide, calcium phosphate monobasic, and a filler agent [9]. Reports show that iRoot BP Plus is biocompatible, insoluble in water, and produces Ca(OH)_2_ when in contact with water [10]. Moreover, iRoot BP Plus has demonstrated significant antibacterial activity against *Candida albicans* and *E. faecalis* [8]. However, it is reported that iRoot BP Plus significantly decreased cell viability of human osteoblasts as compared to MTA after 48 h of exposure [6].

Although MTA and iRoot BP Plus have been widely applied clinically, there is still no consensus regarding the biocompatibility and antibacterial activity of the two materials. Insufficient research has been conducted on the antibacterial activity against *E. faecalis* and the osteogenic differentiation of human periodontal ligament stem cells (hPDLSCs) of these materials, especially when used as retro-filling materials. Therefore, the purpose of this study was to completely assess the biocompatibility and antibacterial activity of MTA and iRoot BP Plus. The results facilitate a deeper understanding of the underlying mechanisms of the biocompatibility and antibacterial activity of these materials for clinical applications.

## Materials and methods

### Cement extracts

MTA (Dentsply Tulsa, OK, USA) and iRoot BP Plus (Innovative Bioceramix, Vancouver, BC, Canada), were prepared according to the manufacturers’ instructions. The materials were molded into discs in the plastic molds (0.8 cm in diameter and 0.2 cm in height) under sterile conditions. The discs were placed in a 5% CO_2_ and 95% air atmosphere for 2 h at 37 °C, washed three times with phosphate-buffered saline (PBS), and subjected to proper ultraviolet irradiation for 30 min. Each material was then socked in aseptic PBS for 2 days; the PBS was replaced every 24 h. Subsequently, the samples were immersed in Dulbecco Modified Eagle’s Medium (DMEM) (Gibco Life Technologies, Melbourne, Australia) for 24 h at 37 °C in a humid chamber. Finally, the eluates were collected and filtered with 0.22 μm filters (Merck Millipore, Billerica, MA, USA). The eluates were diluted at 1:1, 1:2, and 1:4 v/v using fresh DMEM.

### Viability of hPDLSCs

The metabolic activity of treated hPDLSCs (ATCC, Manassas, VA, USA) was determined by 3-(4,5-dimethylthiazol-2-yl)-2,5-diphenyltetrazolium bromide (MTT Cell Growth Kit, Chemicon, Rosemont, IL, USA). The third generation hPDLSCs were cultured in 96-well plates (1 × 10^3^ cells/well) for 24 h in a complete medium. Subsequently, the medium was replaced by the diluted eluates (100 μL/well) prepared previously. The control group’s cells were cultured in the medium without eluates. At 24 and 48 h, 10 μL of MTT was added to each well and cells were continuously cultured for 4 h. Then, the supernatant was aspirated and 100 μL of dimethyl sulfoxide was added. A microplate reader (VersaMax Microplate reader, Sunnyvale, CA, USA) was used to measure the absorbance at 490 nm. The relative proliferation rate was calculated as (assay group/control group) * 100%.

### Analysis of apoptosis and necrosis by flow cytometry

Cell apoptosis and necrosis was evaluated using Annexin V-PE/7-AAD staining [11]. The third generation hPDLSCs incubated with various eluates (undiluted) for 72 h were collected with centrifugation, and resuspended to 2 × 10^5^ cells/mL. The cells were stained with PE conjugated annexin-V and 7-AAD (Immunostep, Salamanca, Spain), according to the flow cytometry protocol for a viability assay. Briefly, 1 μg/mL 7-AAD fluorescent dye was added to the cell suspensions, incubated for 10 min at 37 °C, and centrifuged for 5 min at 800 rpm and 4 °C. The supernatant was removed; 1 mL of PE dye solution was added to the cells, stained in the dark for 15 min, and then filtered. The stained cells were analyzed using a ACEA NovoCyte flow cytometer (Agilent, Lexington, MA, USA). Percentages of live, early, late apoptotic, and necrotic cells were determined by the flow cytometer by using cells cultured in medium without any experimental eluate as control.

### In vitro osteogenic differentiation assay

The third generation hPDLSCs were cultured in six-well plates (1 × 10^5^ cells/well) placed in an incubator for 24 h at 37 °C and 5% CO_2_. Then, the medium was replaced by the extraction medium prepared previously. Cells cultured in medium without any experimental eluate were used as the control. The eluates changed every 3 days. After 3, 7, and 11 days, cells and supernatant were collected for subsequent tests.

#### Real-time reverse transcription polymerase chain reaction (real-time RT-PCR)

Real-time RT-PCR was used to quantify messenger RNA (mRNA) levels of alkaline phosphatase (ALP), collagen type I (COL1), osteocalcin (OCN), Runt-related transcription factor-2 (RUNX2), and Osterix(OSX). Total RNA was isolated from each culture using the RNEasy kit (Qiagen, Valencia, CA, USA) and used as a template for the reverse-transcriptase reaction. The cDNAs were used for templates for real-time RT-PCR. The ALP, COL1, OCN, RUNX2, and OSX gene sequences of hPDLSCs were retrievable from the NCBI database, and utilized for constructing their respective upstream and downstream fragment primer pairs (Table 1). Glyceraldehyde-3-phosphate dehydrogenase (GAPDH) was included as an internal reference gene. The SYBR Green PCR kit (Qiagen) was used to perform real-time RT-PCR. The Ct value indicated the number of PCR cycles needed to gain a certain level of fluorescence. The data were calculated using formula 2^(−ΔΔCt)^.

**Table 1:** List of primers used for real-time RT-PCR

Genes	Primers	Sequence (5′-3′)	dsDNA
ALP	Forward	AGAATCAGAACCACAGGACGGG	22
	Reverse	TTCAAGTCACCTGGGCAAATG	21
COL1	Forward	GCGAGAGCATGACCGATGGATTC	23
	Reverse	GCCTTCTTGAGGTTGCCAGTCTG	23
OCN	Forward	CCCTCACACTCCTCGCCCTATT	22
	Reverse	GGTCAGCCAACTCGTCACAGTC	22
Osterix	Forward	ATCCAGCCCCCTTTACAAGC	20
	Reverse	TAGCATAGCCTGAGGTGGGT	20
RUNX2	Forward	ACCAGCAGCACTCCATATCTCTACT	25
	Reverse	CTTCCATCAGCGTCAACACCATCA	24
GAPDH	Forward	TCAAGAAGGTGGTGAAGCAGG	21
	Reverse	GCGTCAAAGGTGGAGGAGTG	20

#### Western blot analysis

The total protein was extracted using an RIPA lysis buffer (Millipore, Billerica, MA, USA). The protein concentration was tested by the bicinchoninic acid Protein Assay Kit (Bio-Rad, Berkeley, Canada). Proteins were separated by 10% dodecyl sulfate, sodium salt-Polyacrylamide gel electrophoresis (PAGE) and then transferred to polyvinylidene fluoride membranes (Millipore) which were then blocked with 5% fat-free milk at room temperature for 2 h before incubating overnight with primary antibodies against ALP, COL1, OCN, RUNX2, OSX, and GAPDH (Abcam, Cambridge, UK) at 4 °C. After washing for 3 times, the membranes were incubated in corresponding secondary antibodies for 1 h. The blotted bands were detected using an enhanced chemiluminescence detection kit (Amersham Pharmacia Biotech, Little Chalfont, UK).

### Antibacterial activity

The antimicrobial activity of the test materials was evaluated by the DCT against *E. faecalis* ATCC 29212 (Manassas). The strains were grown in tryptic soy agar (TSA, Merc, Germany) at 37 °C for 18–24 h in a 5% CO_2_ and 95% air atmosphere, and collected for subsequent experiments.

A total of 0.05 g MTA or iRoot BP Plus was evenly coated onto the bottoms of the wells of a 96-well plate. Each material was coated with 9 wells, which were divided into three groups with three wells in each group. Samples tested at 20 min, 1 day, and 7 days after mixing (MTA) or addition of the sterile distilled water (iRoot BP Plus) were designated as “fresh group”, “1 day group”, and “7 day group”, respectively [8, 12]. A 10 μL *E. faecalis* suspension (approximately 1 × 10^6^ cells) was added to each well. After incubation in a moist atmosphere at 37 °C for 1 h, 100 μL tryptic soy broth (TSB, Merc) was added to each well. The solutions were mixed for 1 min, the obtained microbial suspensions were diluted tenfold in TSB to 10^−3^. After incubation at 37 °C for 48 h, the number of colonies were counted and expressed as log10 values. *E. faecalis* suspensions added to wells without any material were used as control.

### Statistical analysis

The values were expressed as the mean ± standard error of three independent experiments. Statistically significant differences were assessed by two-way analysis of variance using the GraphPad Prism 8.0.2 software (GraphPad Prism Software, La Jolla, CA). Values of *p* < 0.05 were considered as statistically significantly different.

## Results

### Proliferation of hPDLSCs in the presence of different eluates

**Fig. 1:**
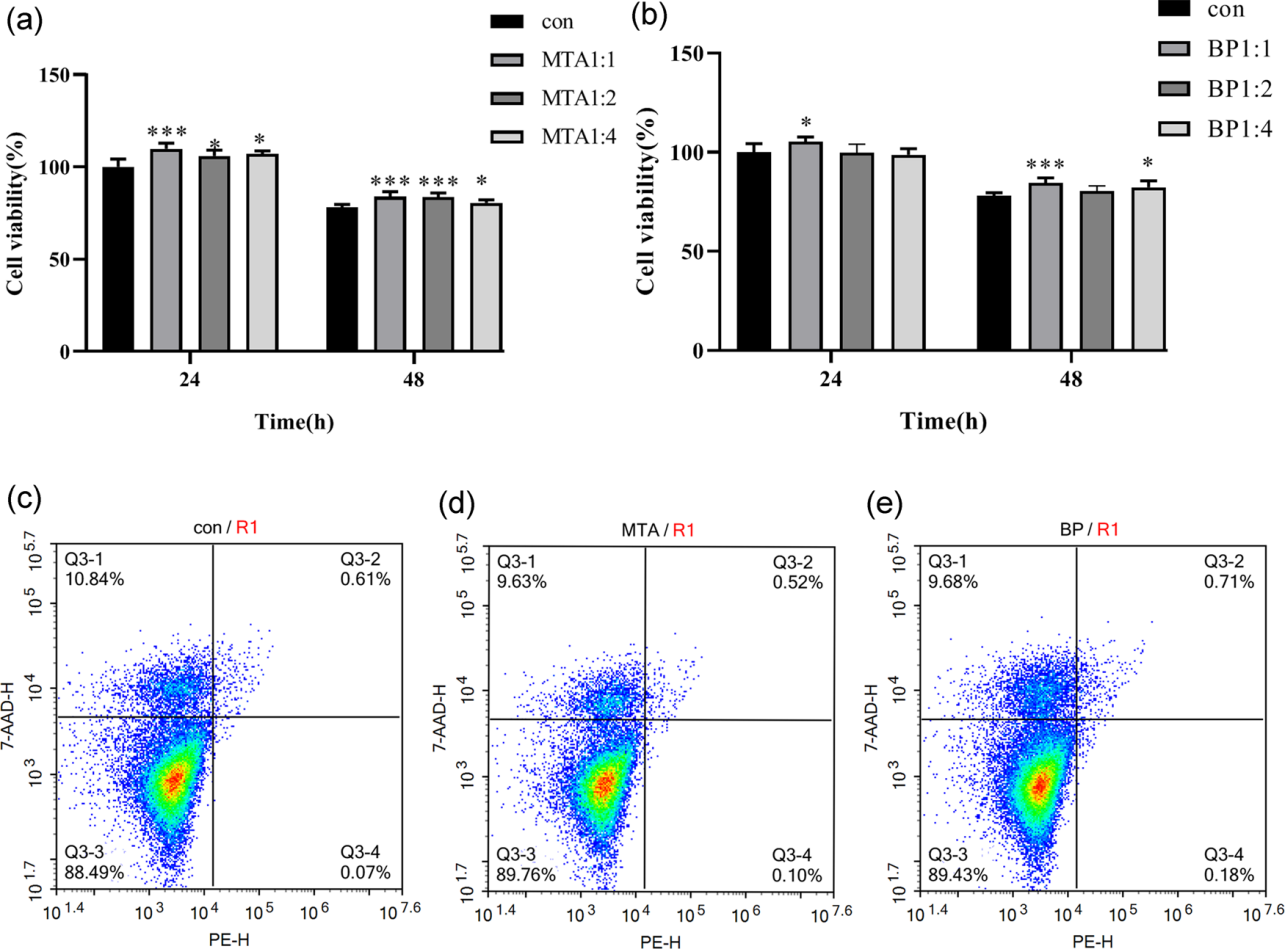
Viability rate of hPDLSCs in different eluates in vitro by MTT assay. **a** hPDLSCs cultured in MTA eluate for 24 and 48 h; **b** hPDLSCs cultured in iRoot BP Plus eluate for 24 and 48 h. (*p < 0.05, **p < 0.01, and ***p < 0.001; *n* = 5). Representative two-dimensional dot plots images of the flow cytometry data from Annexin V-PE and 7-AAD stained hPDLSCs in: **c** control; **d** MTA; and **e** iRoot BP Plus. The dot plots in the three images indicate the distribution of life (lower left), early (lower right) or late apoptotic (upper right), and necrotic cells (upper left)

To determine the influences of both materials about the proliferation of hPDLSCs, the cell viability of hPDLSCs in the eluates of the two materials was measured with the MTT assay (Fig. 1a, b). hPDLSCs of MTA and iRoot BP Plus (dilution 1:1) showed significantly higher cell viability at 24 h and 48 h than that of the control group (*p < 0.05, ***p < 0.001). However, 1:2 and 1:4 dilutions of iRoot BP Plus had an insignificant effect on cell viability (Fig. 1b). Both materials with 1:1 dilutions showed higher values than those with 1:2 and 1:4 dilutions. At 24 h, the cell viability of MTA was significantly higher than that of the iRoot BP Plus group for all dilutions (*p < 0.05); however, no significant difference between both materials was observed at 48 h (*p* > 0.05).

### Apoptosis and necrosis of hPDLSCs in the presence of different eluates

The two-dimensional dot plots shown in Fig. 1c–e exhibited the distribution of live (Annexin-V^−^/7-AAD^−^), early (Annexin-V^+^/7-AAD^−^), late apoptotic (Annexin-V^+^/7-AAD^+)^ and necrotic cells (Annexin-V^−^/7-AAD^+^) in hPDLSCs that were exposed to the eluates of tested materials. The percentage of live cells cultured for MTA and iRoot BP Plus eluates after 72 h (>89%) were slightly higher than that of the control group (88.49%), although this difference was not statistically significant (*p* > 0.05). The percentage of necrotic and apoptotic cells with both materials were similar to those of the control.

### In vitro osteogenic differentiation assay

#### Real-time RT-PCR analysis

**Fig. 2:**
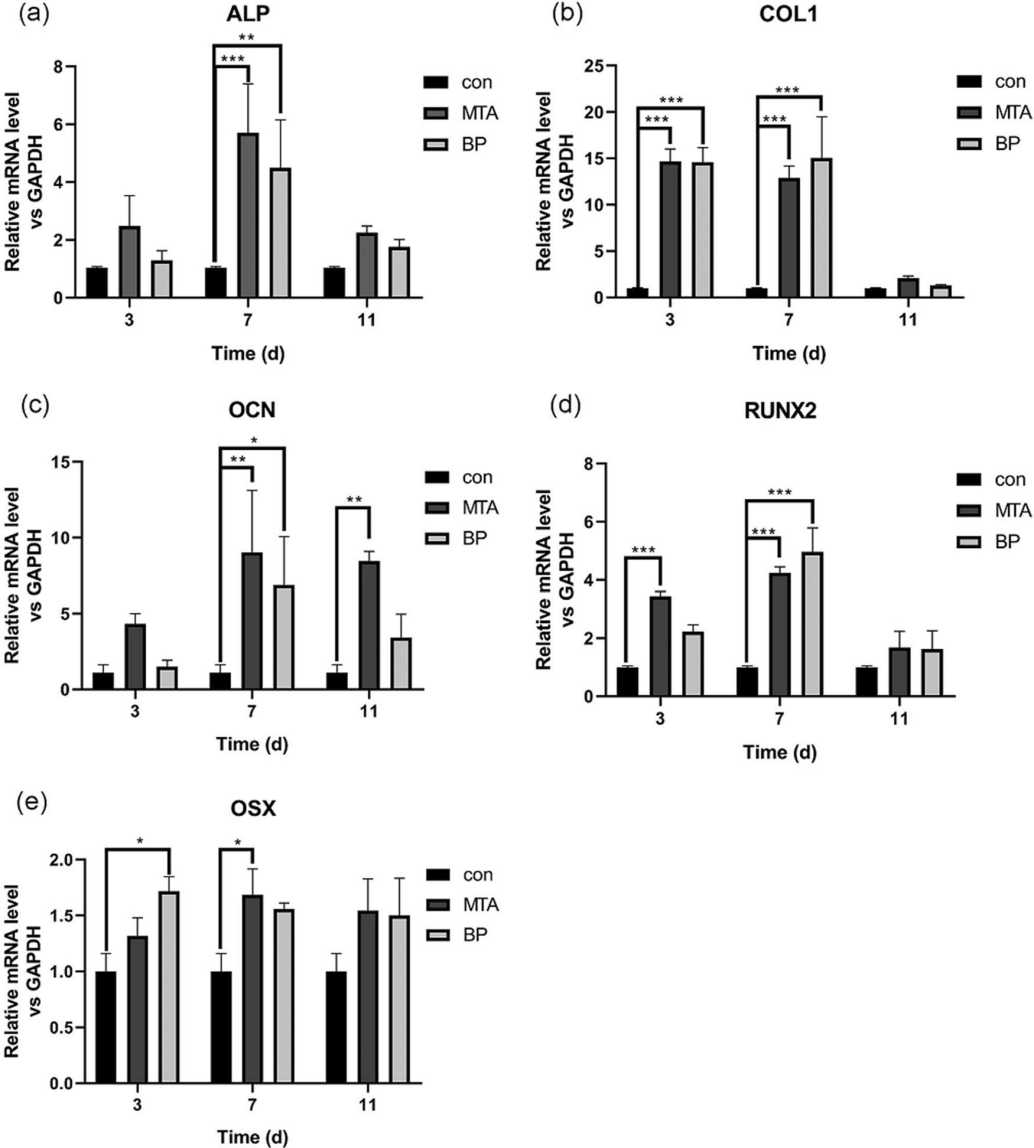
mRNA expression levels of ALP (**a**), COL1 (**b**), OCN (**c**), RUNX2 (**d**), and OSX (**e**) of hPDLSCs with extracts of MTA and iRoot BP Plus, determined by real-time RT-PCR at three time-points (day 3, 7, and 11). (*p < 0.05, **p < 0.01, and ***p < 0.001; *n* = 5)

To investigate the ability of osteogenic differentiation of hPDLSCs treated with root-end filling materials, the levels of mRNA expression of the osteogenic differentiation markers (ALP, COL1, OCN, RUNX2, and OSX) were assessed (Fig. 2). On day 7, the mRNA expression of ALP in both MTA and iRoot BP Plus groups was higher than those on day 3 and day 11, and significantly higher than those in the control group on day 7 (**p < 0.01, ***p < 0.001). Meanwhile, the mRNA expression of COL1 in the two groups on day 3 and day 7 were higher than those in the control group (***P < 0.001), although no significant difference compared with control group on day 11 were observed. On day 7, both experiment groups exhibited significantly higher mRNA expressions of OCN and RUNX2 than those of the control group (*p < 0.05, **p < 0.01, ***p < 0.001).

The mRNA level of OSX in the iRoot BP Plus group was significantly higher than that of the control group on day 3 (*p < 0.05). Although, on day 7, a significantly higher OSX mRNA expression was observed in the MTA group than that of the control group (*p < 0.05). However, mRNA levels of all genes showed insignificant difference between MTA and iRoot BP Plus groups throughout the studied timeframe.

#### Western blot analysis

**Fig. 3:**
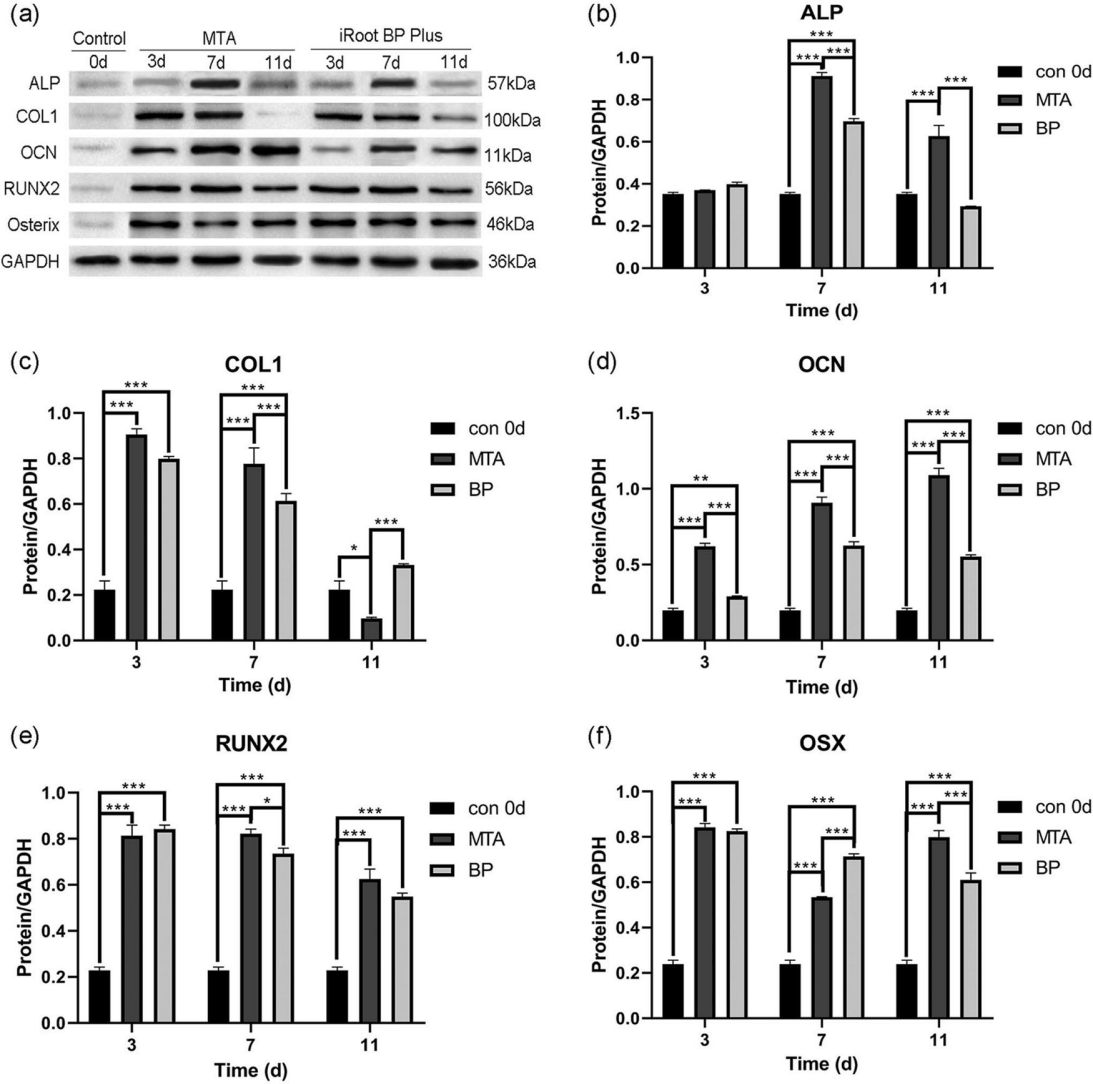
**a** Protein expression levels of ALP, COL1, OCN, RUNX2, and OSX of hPDLSCs with extracts of MTA and iRoot BP Plus, determined by Western blot at three different time-points (day 3, 7 and 11). GAPDH served as an internal control. **b-f** Grayscale analysis of **a**. (*p < 0.05, **p < 0.01, and ***p < 0.001; *n* = 5)

The Western blot assay was used to investigate the protein expression levels of the osteogenic differentiation markers ALP, COL1, OCN, RUNX2, and OSX (Fig. 3). Western blot analysis exhibited that the protein expression of all markers of hPDLSCs in both MTA and iRoot BP Plus groups were significantly higher than those of the control group on days 3, 7, and 11 (**p < 0.01, ***p < 0.001), except for the marker ALP on day 3 (Fig. 3b). The protein expression values of all markers in the MTA groups were significantly higher than those in the iRoot BP Plus (*p < 0.05, ***p < 0.001), except for COL1 on day 11 (Fig. 3c). On day 7, the protein expression of ALP and OCN in both groups were significantly higher than that on day 3 (***p < 0.001). On the contrary, the expression of COL1 in the MTA and iRoot BP Plus groups showed a significant reduction on day 11 (***p < 0.001).

### Antibacterial activity of MTA and iRoot BP plus against *E. faecalis*

**Fig. 4:**
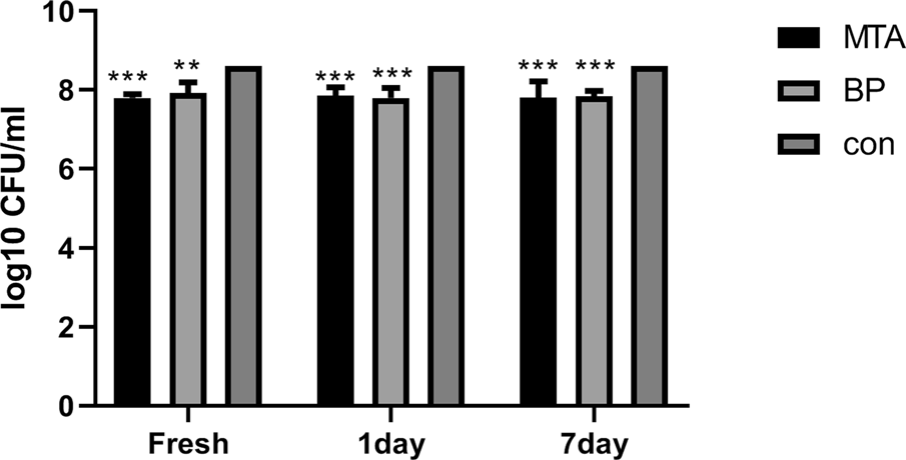
Survival of *E. faecalis* after incubation with MTA and iRoot BP Plus at different experimental periods using the direct contact test. (**p < 0.01, and ***p < 0.001; *n* = 5)

The results of the DCT for the the fresh, 1 day and 7 day groups with *E. faecalis* are shown in Fig. 4. All MTA and iRoot BP Plus groups indicated similar inhibition of *E. faecalis* and statistically significant differences were observed compared with those of the control (**p < 0.01, ***p < 0.001).

## Discussion

A root-end filling material should have the highest biocompatibility because it is in close contact with periapical tissues [13]. The interactions between the stem cells of periapical tissues and root-end filling materials have been considered as important factors because they promote healing [14]. The ability of these cells to form cementum, alveolar bone, periodontal ligaments, nerves, and blood vessels has been demonstrated successfully in vivo [15]. It is widely recognized the interactions between dental stem cells and bioactive materials are very significant in root-end filling applications [16]. Therefore, hPDLSCs were used herein to simulate the close proximity between the tested materials and the apical and periodontal tissues.

In this study, cellular analysis was performed using the eluates from both tested materials. According to the International Standard ISO 10993-12 Biological Evaluation of Medical Devices [17], we obtained eluates of the materials, and hPDLSCs were treated with different eluates, similar to previously reported studies [18–20]. The tests performed on those eluates allowed both qualitative and quantitative assessment of biocompatibility. It could simulate the clinical conditions: the interaction between hPDLSCs and retro-filling materials in blood clots and body fluids.

In vitro cytotoxicity assays are suitable to evaluate basic biological aspects relative to biocompatibility and are simple, reproducible, and cost-effective [21]. Although the results of the MTT assay varied according to dilution and time, both materials could significantly improve the viability of hPDLSCs, showing similar cell compatibility. This result was in good agreement with a previous report on iRoot BP biocompatibility [10]. However, this was not consistent with the results obtained by Zhang et al. [22]; they described the proliferation suppression of human dental pulp cells (hDPCs) in the MTA group during the whole intervention time periods. This discrepancy may be explained because the materials used by Zhang et al. [22] were placed directly in contact with hDPCs, rather than intervening through the material eluates. The results of flow cytometry analyses revealed that neither MTA nor iRoot BP Plus induced apoptosis, thus preserving cell viability. This was consistent with previous reports describing negligible in vitro cytotoxicity of Pro Root MTA and iRoot BP Plus [23].

Periapical bone tissue defects caused by chronic periapical periodontitis can be healed by endodontic therapy or apical surgery [24]. Therefore, the root-end filling materials must have an excellent capability to promote the regeneration of hard tissues. To explore the influences of both materials on the osteogenic differentiation of hPDLSCs, a series of genes and proteins related to osteogenesis were used in this study. ALP is a representative marker of osteoblasts that directly reflects their activity; it is often used as a marker of early osteogenic differentiation and mineralization [25]. OCN is a noncollagenous protein, which regulates the mineralization of hard tissues; it is considered as a marker of terminal differentiation in bone regeneration [26]. RUNX2, involving all stages of biomineralization, is up-regulated in the early stages and down-regulated during the terminal phase and it is also active in mature osteoblasts [27, 28]. OSX is a downstream signaling gene of RUNX2, which is an important transcription factor in osteoblast differentiation [25]. Consequently, the ratio of RUNX2-to-OSX is often considered as an early osteogenic differentiation marker. For the two materials evaluated in this study, the mRNA expression of ALP, RUNX2, OCN, and OSX were upregulated, which was similar with the results obtained by Lu et al. [29]. Meanwhile, we found that the MTA group exhibited significantly higher protein expressions of ALP, RUNX2, OCN, and OSX than those of the iRoot BP Plus group. These data indicated that both materials triggered osteogenic differentiation of hPDLSCs, with MTA exhibiting a superior capacity. Our results indicated the expression of COL1 was downregulated on day 11 for both materials groups. Widbiller et al. [30] reported that COL1, as an early related marker, was down-regulated during the odontoblast maturation process. Hence, we hypothesize that both materials can also induce odontogenic differentiation of hPDLSCs. However, more genes related to odontogenic differentiation need to be tested to confirm this hypothesis.

*E. faecalis* is one of the most detected microorganisms in persistent apical periodontitis [31]. This gram-positive facultative anaerobe exists inside the root canal, and is related to periradicular lesions; it can penetrate and colonize dentinal tubules [32]. For these reasons, *E. faecalis* was selected in this study to evaluate the antimicrobial activity of the materials tested.

For antimicrobial analysis, the direct contact test we performed is a quantitative and reproducible assay that relies on the direct contact of the test microorganisms with test material for a controlled period time; further, this test is independent of the diffusion and solubility properties of the material tested and media [5]. In addition, this method allows the exact measurement of surviving bacteria. The direct contact test can simulate the contact of microorganisms with retro-filling materials in the retrograde cavity of resected roots. Both materials showed significant and similar antimicrobial activities. This result was in good agreement with the conclusions of other studies [8, 33]. Both MTA and iRoot BP Plus produce a calcium silicate hydrogel and Ca(OH)_2_ when in contact with water of the dentin and periapical tissues [8]. Scientific reports indicate Ca(OH)_2_ alters the wholeness of cytomembrane and the transport of bacteria nutrients [34]. Additionally, the alkalizing effect of Ca(OH)_2_ could inhibit significantly the bacterial normal metabolism, growth, and cellular division [35]. Moreover, antimicrobial activity of zirconium oxide against both gram-positive bacteria via redox reactions has been reported in literature [36, 37]. Although several studies have reported the antimicrobial activity of MTA, the results are not consistent [5, 38]. This might be attributed to the use of different experimental methods, bacterial strains, and aerobic or anaerobic culture conditions.

## Conclusion

The novel ready-to-use root-end filling material, iRoot BP Plus exhibited excellent in vitro biocompatibility with hPDLSCs because it promoted proliferation of cells without inducing apoptosis. Both MTA and iRoot BP Plus showed significant as well as similar antibacterial activity against *E. faecalis*. Moreover, both materials could significantly improve the osteogenic differentiation of hPDLSCs. Therefore, both calcium silicate-based materials could be promising root-end filling materials. We believe that further in vivo investigation of these oral biomaterials is required in the future.
